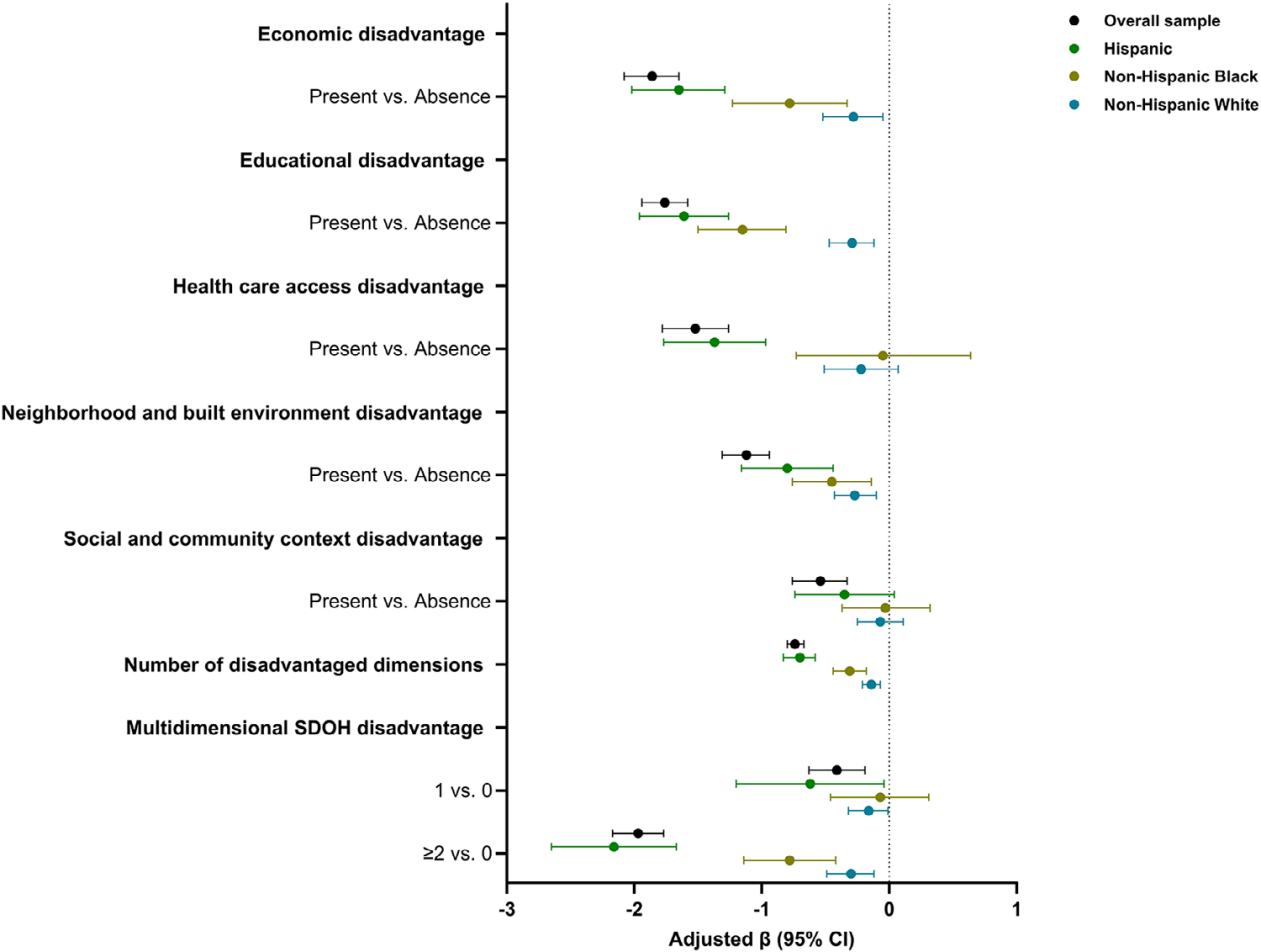# Associations between social determinants of health (SDOH) and cognitive performance among cognitively unimpaired individuals, the HABS‐HD Study

**DOI:** 10.1002/alz70860_096397

**Published:** 2025-12-23

**Authors:** Shanshan Wang, Uyen‐sa Nguyen, Zhengyang Zhou, Sid E. O'Bryant, Kristine Yaffe, Leigh A. Johnson, Rajesh Nandy, The Health and Aging Brain Study (HABS‐HD) Study Team

**Affiliations:** ^1^ University of North Texas Health Science Center, Fort Worth, TX, USA; ^2^ Institute for Translational Research, University of North Texas Health Science Center, Fort Worth, TX, USA; ^3^ Department of Psychiatry, University of California San Francisco, San Francisco, CA, USA; ^4^ Department of Neurology, University of California, San Francisco, San Francisco, CA, USA; ^5^ University of California San Francisco / San Francisco VA Medical Center, San Francisco, CA, USA

## Abstract

**Background:**

The study aims to examine the associations of social determinants of health (SDOH) with cognitive performance among cognitively unimpaired individuals, and to assess whether the associations vary among different racial/ethnic groups.

**Methods:**

We used data from the Health & Aging Brain Study‐Health Disparities (HABS‐HD) study. 2294 cognitively unimpaired participants were included. Multiple dimensional SDOH disadvantages [1) economic disadvantage, 2) education disadvantage, 3) health care access disadvantage, 4) neighborhood disadvantage, and 5) social disadvantage] were defined based on 16 SDOH indicators: 1) household income, 2) occupation, 3) retirement status, 4) education attainment, 5) health insurance, 6) primary care physicians, 7) residence/housing, 8) Area Deprivation Index (ADI), 9) marital status, 10) social support, 11) chronic stress burden, 12) time living in the US, 13) potential nativity, 14) primary language, 15) bilingualism, and 16) acculturation level. Multidimensional SDOH disadvantage refers to the presence of more than one disadvantaged SDOH dimension. The cognitive performance was evaluated by the Mini‐Mental State Examination (MMSE). MMSE score ranges from 0 to 30, with a lower score indicating lower global cognitive function. Linear regression models, with adjustment for age, sex, and APOE4 positivity, were applied.

**Results:**

Dimensional SDOH disadvantages were associated with lower MMSE score among cognitively unimpaired participants (*p* values <0.05) (Figure 1). However, the associations between social disadvantage and global cognition were no longer significant when stratified by race/ethnicity (*p* values>0.05). In addition, the associations between global cognition and economic disadvantage, educational disadvantage, and neighborhood and built environment disadvantage, healthcare access disadvantage were stronger among Hispanic participants. Dose‐response associations with global cognition were observed, with stronger associations among Hispanic participants.

**Conclusion:**

The strength of the associations between dimensional SDOH disadvantages and cognitive performance varies among unimpaired racial/ethnic groups, with stronger associations observed among Hispanics. This may suggest opportunities for early intervention and prevention in this group.